# Hypochlorous Acid (HOCl) as a Promising Respiratory Antiseptic

**DOI:** 10.3390/v17091219

**Published:** 2025-09-07

**Authors:** Michael Winter, Dirk Boecker, Wilfried Posch

**Affiliations:** 1 Team Winter Kompetenztraining, 1030 Vienna, Austria; michael.winter@hocl.at; 2 TOTO Consulting LLC, Campbell, CA 95080, USA; dirk@totoconsulting.net; 3 Institute of Hygiene and Medical Microbiology, Medical University of Innsbruck, Schöpfstrasse 41/R311, 6020 Innsbruck, Austria

**Keywords:** HOCl, respiratory infection, antiseptic, antimicrobial treatment, RSV, influenza, COVID-19, SARS-CoV-2

## Abstract

The COVID-19 pandemic has inflicted unprecedented pressure on communities and healthcare systems around the world. An outstandingly broad and intensive investigation of possible therapeutic interventions is currently taking place to prevent similar future threats to the global population. Investigating the related mechanisms of action is often complex and time consuming. Moreover, research on biochemical interactions of new drugs involves a considerable amount of effort, consequently bearing inherent financial and operational risks for pharmaceutical companies. An interesting approach to counteract colonization and infection is the concept of antiseptic treatment in vivo. Antiseptics are cost-effective and globally accessible, due to their ease of production, transportation and handling. A broad spectrum of active agents with different properties is readily available. One of these substances is hypochlorous acid (HOCl), which is also a naturally occurring biocidal agent and as such part of the innate immune system. Its successful history of medical use in wound treatment, combined with low cytotoxicity and documented efficacy against various pathogens, suggests that HOCl might be an effective agent for treating the respiratory mucosa. This could potentially enable therapeutic inhalation for combating bacterial infections and viral pathogens such as human respiratory syncytial, influenza, and SARS-CoV-2 viruses, which will be discussed in the present article.

## 1. Introduction

The pursuit of novel antibiotic treatment options to mitigate the worldwide problem of growing bacterial resistance has become increasingly difficult and cost-intensive in recent years [1]. Also, the efficacy of antibiotics and other pharmacological interventions mostly depends on the correct diagnosis of the underlying pathogen.

In comparison, an antiseptic approach is limited to mainly external application, but more resilient against diagnostic ambiguities and errors, due to its agnostic mechanism of action [2,3].

Its broad efficacy is therefore advantageous in less developed areas with limited access to medical infrastructure. Concurrently, in suitable fields of application it may prove helpful even in advanced medical institutions by exerting protective action against various nosocomial pathogens [4], with the possible benefit of containing resistance development.

A variety of existing antiseptic agents exhibit notable resilience against microbial resistance, characterized by their continuously effective reduction in bacterial loads in vitro [5,6]. Due to their broad spectrum of action, antiseptic treatments also provide the advantage to be utilized against viral and fungal infections [7,8].

Thus, especially considering emerging viruses and the COVID-19 pandemic, it would be pertinent to investigate the potential applications of these antiseptic agents in treating common infections, like those affecting the respiratory mucosa. Understanding their effectiveness in such contexts could provide valuable insights into expanding their therapeutic utility beyond conventional uses.

However, in a potential new scientific field of antiseptic treatment of respiratory tract infections, it is crucial to rigorously evaluate safety concerns. For these efforts it is reasonable to re-examine the respiratory pathways, for their distinct tissue properties, characteristics in microbiome and systemic functional contributions. Depending on the area of mucosal application, a variety of different antimicrobial and antiviral agents may be considered [9,10].

## 2. Antiseptics of Interest

In their recommendations in the context of the COVID-19 pandemic [11], the German Society for Hospital Hygiene (DGKH) mentioned various antiseptic agents, including two pre-exposure prophylactic agents used at the University Medicine Greifswald, povidone-iodine (PVP-I) and essential oils:

In the area of medical antiseptics, povidone-iodine (PVP-I) has been used internationally in tolerable concentrations between 0.23% and 1.25% inside the nose and mouth for prophylaxis, and could be effective in early treatment [11,12]. A mouthwash with essential oils is commercially available with and without ethanol, and has achieved relevant log reductions in vitro [11,13]. For sensitive individuals it is possible to gargle with green tea or aronia juice, combined with the use of saline solutions or carragelose [14] for the nasal passages.

However, not all common antiseptics were reported as effective against SARS-CoV-2. Common antiseptic mouthwashes based on hydrogen peroxide (H_2_O_2_), polyhexanide and octenidine were described as not sufficiently effective against SARS-CoV-2 in vitro, but remain an interesting alternative for other types of infection [10,11].

The DGKH outlined its perspective on sodium hypochlorite and HOCl. A comparative in vitro study with encouraging results of an HOCl/stabilized hypochlorite mouthwash was mentioned [11,13]. Also, a combination of sodium hypochlorite (NaOCl) and HOCl with a gel matrix achieved a log reduction of 2.21 within 30 s, rendering it the only nasal spray with a potentially clinically relevant log reduction in SARS-CoV-2 in another comparative study [11,15]. The authors recommended it as a post-exposure prophylaxis, but suggested that tolerability of long-term use of NaOCl needs clarification.

Although not mentioned in the DGKH paper, the HOCl derivative N-chlorotaurine (NCT) formed by reaction with taurine might be considered as well, and has been suggested to be a suitable agent for respiratory disinfection [16].

While several agents were discussed in the DGKH publication, HOCl may be considered particularly interesting, due to its natural presence in the respiratory tract [17] and its reported efficacy.

## 3. HOCl

In general, an ideal antiseptic agent should possess attributes such as low cell toxicity, sufficient efficacy against a broad spectrum of pathogens, and should be non-allergenic as well as non-irritating. Current research demonstrated all of these favorable characteristics for HOCl [3,18], a compound integral to the innate immune system.

### 3.1. Key Role of HOCl in the Innate Immune System

White blood cells, particularly neutrophils and monocytes, produce reactive oxygen species like H_2_O_2_ and HOCl upon cell activation. This can be induced through the activation of PRRs like Toll-like receptors, Fc receptor binding and several other mechanisms [19].

HOCl is a weak acid but a strong oxidizing agent. It interacts with structural components of viruses and bacteria, making it a crucial endogenous molecule for effective phagocytosis [3]. Macrophages and microglia cells produce HOCl from intracellular NaCl using the myeloperoxidase enzyme system. The reactive HOCl is then released to oxidize the invading pathogen, and ultimately converted back to NaCl [20].

In biological systems, HOCl can be regarded as the most effective and most common oxidizing agent. It is one of the foremost endogenous molecules for effective phagocytosis of invading bacteria or viruses [21].

### 3.2. Production of Exogenous HOCl

HOCl has been broadly used in therapeutic applications, as well as for disinfection of surfaces, water, food and room air [22]. Different production methods of HOCl have been established, to meet the requirements of their respective industrial use.

HOCl solutions can be produced through 2 methods: either direct injection of gaseous chlorine into water, or electrolysis of hydrochloric acid or an aqueous solution of sodium chloride. Several factors influence the production and efficiency of electrolyzed water, including water and electrolyte properties, electrode material, current, storage, and application factors. A comprehensive overview is given by Ampiaw et al. [23].

In aqueous solutions under physiological conditions the pair of HOCl with its conjugated base OCl^−^ represents the potent oxidizing redox system [E0’ = +0.9 (OCl^−^); E0’ = +1.48 V (HOCl)]. At a pH of about 7.5, the concentration of HOCl and its correlated base form OCl^−^ are approximately equal [24,25].

At pH 5 HOCl is the dominant oxidizing agent. Lowering the pH of HOCl/OCl^−^ solutions below 5 increases the oxidation potential monotonically, caused by a proportional increase in the Cl_2_ (aq) concentration (extensively studied via Raman spectroscopy by Cherney et al. [26]).

### 3.3. Reaction Mechanisms of Externally Applied HOCl

In wound treatment, HOCl at suitable concentrations supports the healing process upon application. Research has demonstrated its beneficial effects on healing mechanisms of the mucosa and other tissue types, rendering it an effective ingredient and possible future gold standard for topical wound care solutions [27].

The chemical reaction of HOCl with endothelial structures deactivates microbes by oxidizing their cellular components, such as proteins, lipids, and DNA, leading to cell death [20]. This reaction is non-specific and effectively kills a wide range of microbes, including bacteria, viruses, and fungi.

Furthermore, HOCl stimulates the production of neutrophil extracellular traps (NETs), which can trap and kill microbes [28,29].

Besides its direct biocidal activities HOCl is also involved in complement-mediated immunity, a synergistic acting protein cascade of the innate immune system that helps eliminate pathogens and initiate inflammation [20,21,30]. HOCl activates immune cells, such as macrophages, recruiting them to the site of infection.

Finally, it promotes the abundant presence of the antimicrobial acting N-chlorotaurine (NCT) in the extracellular medium. This reversible reaction modulates the oxidizing capability of HOCl through the formation of the less reactive oxidant NCT [31].

Due to these antimicrobial properties, HOCl is expected to exert significant influence on the microbiome of the application area. Therefore, it is important to investigate possible adverse systemic effects of its use on the respiratory mucosa, particularly in emerging applications such as inhalation.

## 4. Systemic Relevance of Respiratory Microbiome Reduction

### 4.1. Mucosal Environment and Reactive Oxygen Species

In healthy individuals, upper airways are relatively robust and typically host a diverse and balanced array of microbial species, collectively known as the mucosal microbiome [32,33].

Changes in its equilibrium can be potentially problematic. Accumulations of specific bacterial organisms are often linked to cardiovascular diseases like hypertension and various forms of dementia, among them *Chlamydia pneumoniae* [34] in the pharynx, or *Treponema denticola* [35] and *Porphyromonas gingivalis* [36] in the oral cavity. Bacterial colonization leading to caries and periodontitis contributes to a broad spectrum of systemic disorders [37].

The metabolic activity of the mucosal microbiome contributes to a variety of essential systemic mechanisms. Some of the factors possibly being impacted by antiseptic intervention are vitally important, like the bioavailability of nitric oxide (NO), through which the mucosal microbiome likely participates in the regulation of blood pressure [38,39] and the sleep–wake cycle [40].

The respiratory epithelium employs mucociliary mechanisms to continually clear inhaled particles and pathogens by constant movement of mucus to the oropharynx and eventual dispatch through the digestive system [41]. Bronchial tissue and its secretions contain a range of immune cells, including dendritic cells, macrophages and highly mobile neutrophils, which all contribute to the defense against respiratory infections by producing HOCl and other reactive oxygen species (ROS), and thus rendering HOCl an inherent part of the mucosal environment [42,43].

Apart from these immune cells, several types of other cells of the ciliated epithelium also recognize bacterial components through specific PRR-receptors on their surface. Activation of these receptors triggers signaling pathways, which leads to the release of inflammatory mediators like cytokines and chemokines [44,45]. One of these cytokines is interleukin-6 (IL-6), which acts as a pyrogen and plays an important role in the immune response during inflammation and infection. In addition to epithelial cells, it is also produced by fibroblasts and macrophages [46].

Epithelial cells contribute directly to the control of microbial contamination, producing antimicrobial peptides, surfactant proteins, immunoglobulins and ROS including HOCl [3,17,47,48].

As a result of the combined effect of this interplay, healthy microbiome of lower airways typically shows similar microbial community variation with significantly lower log counts [49]. Its metabolic products likely exert only minor effects on systemic functions.

### 4.2. Implications for Inhalation

Inhalation of supplementary exogenous HOCl is anticipated to have minimal impact on oral microbial biomass compared to mouthwashes, due to its minor concentration and dosage. In the lower airways, microbial counts are generally low. Additionally, antiseptic treatments are generally less specific and therefore less likely to inflict dysbiosis than current antibiotics [50]. Therefore, regarding the respiratory microbiome, antiseptic inhalation is not expected to pose greater concerns than established antibacterial treatments such as antimicrobial mouthwashes targeting the pharyngeal mucosa.

It may instead be anticipated to provide a favorable effect in the respiratory tract similar to toothpaste [51] and some mild antiseptic mouthwashes [52], evening out dysbiosis while counteracting infection.

## 5. HOCl-Containing Products

An increasing variety of HOCl-containing health products is available throughout Europe. Depending on their intended use, they are predominantly approved as medical devices in the appropriate class, as shown in Table 1.

In the United States, several medical devices formulated with HOCl have FDA 510(k) clearance [53,54]. Some HOCl-based products have entered dermatology [55], and more recently the consumer cosmetics market, receiving growing public attention [56].

**Table 1:** Examples of HOCl-based medical devices and health products in Europe.

Product Name	Manufacturer	Country	Total Free Chlorine (ppm)	Medical Device Class	Approval	Recommended Use
Plasma Liquid^®^ Nasal Spray	Regeno GmbH	Mannheim, Germany	<600 [57]	IIa	CE marked	Cleansing and moisturizing the nasal mucosa
Granudacyn^®^ Mouth Wash	Mölnlycke Health Care GmbH	Düsseldorf, Germany	105 [58]	IIa	CE marked	Mouthwash for oral hygiene and pathogen reduction
Oji Biosafety Room Air Disinfection System	oji Europe GmbH,	Nauen, Germany	<0.5 (regulatory limit [59], *)	not applicable	not applicable	Long-term air disinfection to reduce airborne pathogens
Veriforte^®^ Wound Irrigation Solution	P.G.F. Industry Solutions GmbH	Elixhausen, Austria	93 [58]	IIb	CE marked	Cleansing and irrigation of acute and chronic wounds
Actimaris^®^ Nasal Spray	ActiMaris AG	Appenzell, Switzerland	440 [60]	I	CE marked	Cleansing and moisturizing the nasal mucosa
Hydroliq Fogging Solution	Hydroliq AG	Lucerne, Switzerland	<0.5 (regulatory limit [59], *)	not applicable	not applicable	Surface and air disinfection in various settings
Microdacyn60^®^ Wound Care Solution	Oculus Innovative Sciences Netherlands B.V.	Delft, Netherlands	80 [58]	IIb	CE marked	Cleansing and irrigation of acute and chronic wounds
Spectricept™ Skincare Solution	SpectrumX Direct Limited	Knutsford, United Kingdom	unknown	IIa	CE marked	Skincare solution for cleansing and moisturizing
OraWize+ Mouthwash/Dental Rinse	Tec-Safe	Maccelsfield, United Kingdom	100–200 [13]	IIa	CE marked	Mouthwash for oral hygiene and pathogen reduction
Briotech Topical Skin Spray	Briotech EU	Vantaa, Finland	unknown	not applicable	unknown	Topical spray for cleansing and moisturizing the skin

* Reference occupational exposure limit for chlorine in workplace air (Germany).

## 6. Dosage and Safety Considerations on HOCl-Inhalation

The concentrations of HOCl vary significantly depending on the intended application. It is important to consider pH and the ratio between HOCl and other ROS in these products, especially those produced through electrolysis, with the goal of ensuring that HOCl, the more effective and less cytotoxic component, is the predominant species [61,62].

In the context of inhalation, recent evidence notably suggests that aerosolized base fluids containing up to ≈5.72 mM (300 ppm) HOCl at a pH of 6.5 do not affect respiratory tissue, cellular viability, or morphology [63,64].

While it is commonly presumed that droplet size of aerosols defines the maximum bronchiolar propagation range, from a physical perspective some outgassing from droplets in their proximity is to be expected during inhalation. Traces of HOCl in its gas phase will therefore likely reach the pulmonary alveoli, along with some very small droplets under 1 μm [65,66]. Alveolar membranes in the human lung are very delicate with a minimum thickness of less than 1 μm [67,68], underlining the diligence of choosing the lowest effective concentration of any oxidizing agents applied.

Biological HOCl concentrations within inflamed tissue can reach micromolar levels of about 200 μM (≈10.5 ppm) [69], contributing to oxidative stress and subsequently host cell damage during chronic inflammation. The mucosa of the respiratory tract and the lung parenchyma counteract excessive ROS damage by a variety of mechanisms, utilizing antioxidant substances like ascorbic acid, α-tocopherol, glutathione and taurine [70,71], in accordance with the complex role played by NO [72].

High second-order rate constants, in the range of 10^7^ to 10^8^ M^−1^·s^−1^, are observed for reactions of HOCl with cysteine and methionine [73], as well as thiocyanate (SCN^−^) [74]. The reaction with SCN^−^ leads to the formation of hypothiocyanite (OSCN^−^), a mild oxidant with synergistic antimicrobial properties [75].

A modest amount of oxidative stress is thought to be beneficial rather than harmful to human cells, inducing a hormetic response [76,77], by which intracellular adaptive mechanisms to low-level stressors may potentially increase cellular lifespan and health span.

Depending on HOCl concentration, different mechanisms of action against pathogens have been discussed. Some authors have proposed that concentrations in the micromolar range impact bacterial DNA synthesis, while concentrations in the millimolar range result in membrane disruption [78,79].

ROS inducers have been suggested as a possible intervention against viral infections, including SARS-CoV-2, by impairment of RNA integrity [80]. On the other hand, oxidative stress also contributes to viral pathogenesis and exerts pro-infectious effects [81]. For example, recent research has proposed enhanced ACE2-binding by SARS-CoV-2 due to oxidative conditions locally facilitating the formation of disulfide bonds [82].

**Figure 1:**
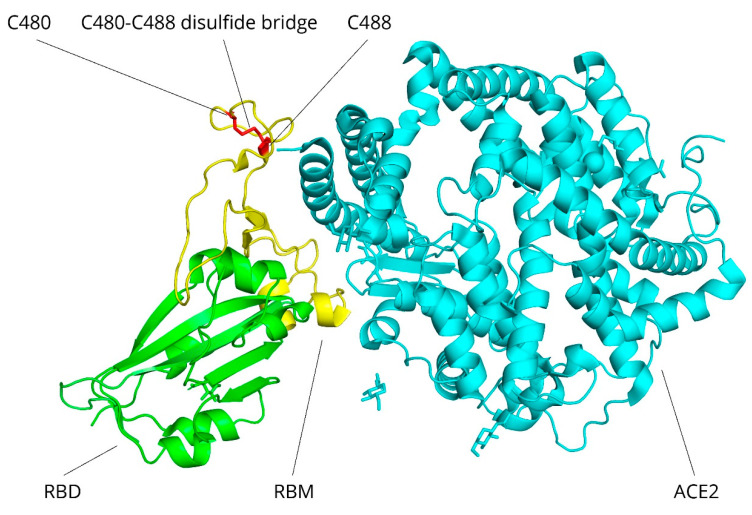
SARS-CoV-2 receptor-binding domain bound to the extracellular domain of the human ACE2-receptor. The receptor-binding domain (RBD) of the spike protein (green) is engaging human ACE2 (cyan) through the receptor-binding motif (RBM, yellow). The cysteine residues C480 and C488 (red), as well as their disulfide bridge, are sterically exposed and critical for ACE2 binding. Structure visualized using PyMOL [86] and based on PDB entry 6M0J [85].

However, HOCl rapidly compromises and cleaves disulfide bonds at very low concentrations [83,84]. This may constitute an interesting virucidal mechanism by external HOCl through rapid cysteine interaction, overoxidation, and cleavage of solvent-exposed disulfide bridges, for example, between cysteine residues C480 and C488 [85] in the SARS-CoV-2 receptor-binding motif (see Figure 1).

Conceivable additional targets within the spike glycoprotein include the accessible cysteine residues and disulfide bond at C391-C525 and the bridge at C379-C432 [87], as well as other reactive residues such as methionine, histidine, tyrosine, and tryptophan [88].

A similar approach to virucidal effects by low supplemental HOCl-concentrations may be feasible in human respiratory syncytial virus (RSV) as well, for example through the oxidation of the structurally critical and sterically accessible residues C69/C212 [89] and C37/C439 [90], as well as cleavage of their respective disulfide bridges, in the RSV F protein (PDB entry 4JHW [91]).

Compared to SARS-CoV-2 and RSV, influenza virions appear to be even more susceptible to oxidation, due to their delicate lipid layer and high dependance on exposed disulfides [92]. Correspondingly, influenza A strains exhibited high vulnerability [93], and less resistance than SARS-CoV-2 [94], in fogging experiments with low-dose HOCl.

For an inhalative intervention, a conservative and practical strategy would involve using concentrations close to physiological levels, aiming for a target concentration of approximately 200 μM. Experimental studies suggest a considerable loss in free chlorine concentration of airborne electrolyzed water during spraying, reaching almost 50% decrease during a travel distance of 25 cm [95].

Furthermore, dilution at the target site, as well as reactive consumption by surfactant proteins, thiol groups and other reactive species is to be expected.

However, under infectious and inflammatory conditions, the buffering capacity of the airway lining fluid may be compromised [96]. In an experimental decontamination effort or early treatment attempt, it therefore seems reasonable to employ a maximum concentration of approximately 500 μM (≈26 ppm) HOCl as the base fluid for aerosolization, and thus aiming for mild antimicrobial effects by targeted oxidation of sterically exposed amino acid residues, rather than classical antiseptic log reduction through membrane disruption.

This strategy involves comparatively low external HOCl concentrations, however it may induce favorable secondary biochemical reactions in situ, which lead to a selectively increased oxidative potential in close proximity of some pathogens (discussed in Section 7). Additionally, physiological temperature levels are expected to significantly enhance HOCl reactivity in vivo and further improve antimicrobial action [97].

## 7. Discussion

The possibility of administering antiseptic treatments repeatedly in quick succession (e.g., 3 or 4 times per day), with the goal of reducing viral and bacterial loads over prolonged periods, may help alleviate the metabolic burden of infection and improve clinical efficacy. This strategy relies to a significant extent on low cytotoxicity and side-effects, which is an established advantage of HOCl in wound treatment [3], since the majority of publications reports low cytotoxicity [98] in wound treatment and no side effects in peritoneal lavage [99].

While other antiseptic agents like benzalkonium chloride or triclosan may induce cross-resistance in some bacterial species, there is no documentation of resistance facilitated by NaOCl [100].

There is some evidence for a “trade-off” between antimicrobial efficacy and undesirable effects like decreasing viability of keratinocytes and fibroblasts [58] when HOCl is applied in vitro in certain millimolar concentrations. However—in vivo concentrations of HOCl are normally in a much lower micromolar range.

While HOCl as an oxidative species may initially have beneficial effects in infections, ROS have been documented to play a role in the formation of macrophage extracellular traps [28], with prolonged excess levels contributing to chronic inflammation. Chronic oxidative stress and inflammation in a typically heterogeneous ROS-dominated milieu is widely acknowledged to be detrimental, can promote viral replication [81], and contributes to aging and oncogenesis [101].

Conversely, excessive ROS levels can also induce cancer cell death, and are deliberately exploited in therapeutic approaches such as radiation and chemotherapy [102]. Thus, accurate distinction of oxidative species, concentration, timing and location is a prerequisite for determining the biological outcome.

Interestingly, HOCl was shown to induce apoptosis selectively in malignant cells expressing NADPH oxidase. Selective action against transformed cells apparently depends on the interaction between their NOX1-generated superoxide anions and HOCl, resulting in hydroxyl radicals, which then induce apoptosis [103].

HOCl has been documented to ameliorate nuclear factor-κB-mediated skin diseases in mice [104], and a solution containing HOCl and NaOCl blocked tumorigenic UV-induced inflammatory progression in another murine model [105,106,107].

Recently, HOCl has also shown favorable results in a comparison of oral antiseptics for radiation- or chemotherapy induced mucositis [108]. Furthermore, a recent in vitro study illustrated reduced binding of IL-6 to its receptors following treatment with HOCl, and this reaction with IL-6 receptors showed high reactivity already at 72 µM [109]. Notably, not just HOCl itself, but also its derivative N-chlorotaurine (NCT) is able to downregulate IL-6-dependent pro-inflammatory pathways, which play an important role in clinical conditions like those seen with severe COVID-19 [16,110].

Further clinical research on the possible role of HOCl in the ongoing fight against SARS-CoV-2 was recommended in several recent publications [3,22,111]. A randomized, double-blind, controlled clinical study is currently ongoing in parts of Europe, inquiring into the potential of HOCl-containing nasal spray and mouthwash as an early treatment against COVID-19. It is evaluated if this combined intervention, several times per day over ten days, can reduce viral load, shorten the duration of test positivity or alleviate symptoms [112].

HOCl has been acknowledged as effective against SARS-CoV-2 in vitro by a Japanese NITE special committee, at concentrations of 35 ppm and above [113]. It also inactivated oral pathogens and a SARS-CoV-2 surrogate within 30 s in vitro at low concentrations (45–60 ppm), even in the presence of saliva. In the same study, further tests with a 26 ppm solution still documented encouraging antimicrobial activity by the complete inhibition of *Prevotella intermedia* [2].

Antiviral efficacy against human norovirus and two surrogate viruses was observed at very low concentrations, with HOCl applied both as a liquid and a fog at 18.8 ppm and above [114].

Efficacy of HOCl against biofilms like those created by *Streptococcus mutans* and *Streptococcus gordonii* was documented [115,116], exhibiting its high oxidative reactivity against proteins and peptide bonds. More specific research on its putative potential to inactivate toxins, like bacterial exotoxins or mucosal complement anaphylatoxins [117,118] by topical application is warranted.

Despite their early documentation [79], the remarkable antimicrobial effects of HOCl in regular biological concentrations of only 50 μM (≈2.6 ppm), including the apparently complete inactivation of the growth of *E. coli* within 5 min in vitro, have not resulted in extensive clinical research to date.

The presence of pathogens correlates with local concentrations of biological ROS. Potential explanations for a higher-than-expected in vivo efficacy of externally applied micromolar HOCl therefore include its possible interaction with locally available biological H_2_O_2_ and, like in the beforementioned anti-oncogenetic effect, interaction with local superoxide anions. This may selectively lead to the formation of singlet oxygen and hydroxyl radicals capable of inducing apoptosis in the close proximity of pathogens [103,119]:

HOCl + H_2_O_2_ → H_2_O + ^1^*O*_2_ + H^+^ + Cl^−^

HOCl + O_2_^⋅−^ → ⋅OH + Cl^−^ + O_2_

## 8. Conclusions

An experimental clinical strategy for early treatment of a broad range of bacterial and viral infections, including those caused by the *Pneumoviridae* family like RSV, as well as influenza viruses and SARS-CoV-2 [8,120,121,122], could be based on inhalation, evaluating aerosolized low-dose HOCl solutions of up to 500 μM (≈26 ppm) in controlled trials.

A conservative approach, loosely resembling and supplementing endogenous countermeasures to infections, may presumably be repeated frequently—initially covering a time period similar to the reaction time of the adaptive immune system, aiming to keep microbial or viral replication and log counts low and manageable, until specific antibodies and T-cells have arrived at the infection site.

Therapeutic success of this kind of intervention would likely result in mitigation of clinical symptoms and reduced morbidity rather than PCR test negativity, since impaired and non-reproductive virions or bacteria [123] will still generate positive test results.
